# The denominator problem: Estimating MSM-specific incidence of sexually transmitted infections and prevalence of HIV using population sizes of MSM derived from Internet surveys

**DOI:** 10.1186/1471-2458-9-181

**Published:** 2009-06-11

**Authors:** Ulrich Marcus, Axel J Schmidt, Christian Kollan, Osamah Hamouda

**Affiliations:** 1 Robert Koch Institute, Dept. Infectious Diseases Epidemiology, PO box 650261, 13302 Berlin, Germany; 2 Social Science Research Center Berlin (WZB), Public Health Unit, Reichpietschufer 50, 10785 Berlin, Germany

## Abstract

**Background:**

Measuring prevalence and incidence of sexually transmitted infections in hard to reach populations like men who have sex with men (MSM) is hampered by unknown size and regional distribution of this population. Community sample – and study-based measurements are often fraught with participation biases and do not allow generalization of the results for other regions or the whole population group of MSM.

**Methods:**

We used the proportional regional distribution of participants of large internet-based surveys among MSM from Germany together with a general population survey-derived estimate of the MSM population to estimate regional population sizes. Based on transmission group category from surveillance data and regional MSM population size we calculated regional population-specific incidence rates of newly diagnosed HIV infection and syphilis. For HIV prevalence we compared estimates of prevalent HIV infections in MSM from a surveillance data-based model with a mixed model in which we used the proportional regional distribution of HIV positive participants from surveys and the estimated total number of prevalent HIV infections from the surveillance based model.

**Results:**

Assuming a similar regional distribution of survey participants and the MSM population as a whole, the regional proportion of MSM in the general population can be estimated. Regional incidence calculated with the estimated MSM population as denominator and national surveillance data as numerator results in regional peak incidence rates of 7–8 per 1,000 MSM for newly diagnosed HIV infection and syphilis. The gradient between metropolitan and rural areas narrows considerably compared with calculations which use the total (male) population as denominator. Regional HIV prevalence estimates are comparable in the two models.

**Conclusion:**

Considering the difficulties to obtain regionally representative data by other sampling methods for MSM, in Western post-industrialized countries internet-based surveys may provide an easy and low cost tool to estimate regional population distributions. With national surveillance data, which categorize transmission groups, regional population-specific incidence rates for reportable sexually transmitted infections can be estimated. HIV prevalence estimates for regional MSM populations show differences related to the level of urbanization, MSM concentration, and starting points of the HIV epidemic in western and eastern Germany.

## Background

Currently there is a lack of simple and reliable methods of measuring the regional prevalence and incidence of health conditions or infections in subpopulations such as men who have sex with men (MSM) because the size of the denominator (total number of MSM) is usually unknown. Prevalence measurement in convenience samples is subject to numerous biases and it is difficult to generalize results to the whole population of MSM or to other regions. Incidence measurement is even more difficult. The most reliable method is direct measurement in longitudinal cohort studies, but cost and necessary infrastructure are prohibitive for widespread use. In addition, the experience from many intervention trials and cohort studies has demonstrated that participation in a study often leads to a reduction of incidence compared to baseline, independent of the interventions even in the control arm of studies.

Because of difficulties in recruiting sufficiently large samples of MSM, previous efforts to estimate subpopulation-specific incidence and prevalence of HIV usually remained restricted to metropolitan areas with enlarged proportions of MSM in the population [1,2]. Prevalence and incidence measurement for rural MSM populations are almost non-existent.

In many western post-industrialized countries the HIV epidemic prompted efforts to collect data on transmission group for surveillance of HIV and selected STIs (in most countries, these groups separate MSM from intravenous drug user, or heterosexual contacts). These surveillance data likely underestimate MSM-related transmission, because the transmission group reported by health care providers requires communication about sexual behaviour between client and care provider and disclosure of often stigmatised behaviour by the client. However, the main problem in using these data for subpopulation-specific diagnosis incidence measurement and prevalence calculation is the unknown size and regional distribution of the MSM population (denominator). Population-based surveys have recently been used to estimate the total size of MSM populations [3,4], which allows the calculation of national prevalence and incidence rates for the MSM population based on surveillance data which report transmission risk. However, it would require very large sample sizes for general population surveys to make reliable estimates for the regional size of small subpopulations like MSM.

### Objectives

We used the regional distribution of (predominantly online) survey participants and an estimate for the total number of MSM in Germany derived from representative studies on the general population to estimate regional population sizes of MSM at the level of federal states and the largest cities [5]. Using these estimates as denominator and HIV and syphilis diagnosis data (attributed to MSM) from the statutory infectious disease surveillance system in Germany as numerator, we calculated MSM specific incidence rates of newly diagnosed HIV infection and syphilis. We compared these subpopulation specific incidence rates with transmission group-specific incidence rates that relate to the general population as denominator. We also explored whether estimates on regional HIV prevalence for MSM based on the regional distribution of HIV positive survey participants lead to comparable results as prevalence estimates based on surveillance data.

## Methods

### HIV and syphilis surveillance data

The German HIV surveillance system has been described in detail elsewhere [6]. Briefly, newly diagnosed HIV infections must be reported anonymously, but with a unique identifier, by laboratories with complementing patient history and clinical data provided by the primary care physician on a duplicate of the laboratory reporting form. The syphilis surveillance system is similarly organized, with the only difference that syphilis is reported without a unique identifier [7]. This requires an extensive search for double reports, based on birth date, postal code and any other matching information on the report forms. Regional allocations are based either on the first three digits of the five digit postal code of the patients' place of residence, or if not provided, on the postal code of the health care provider, or if this is also missing, on the postal code of the laboratory. For the years 2006/07 information on transmission group was available for 73% of the syphilis reports and 86% of reports of newly diagnosed HIV infections. For HIV incidence analysis it was assumed that reports without information on transmission risk have a risk distribution equal to cases with risk information. For syphilis surveillance data, in which reported transmission group categories are heterosexual, homosexual, and unknown/no risk identified, we counted all male patients as MSM unless heterosexual transmission was explicitly reported. By doing so, 323 females and 361 males could be attributed to heterosexual contacts in 2006, and 263 females and 352 males in 2007. 2,467 cases were counted as MSM in 2006 and 2,629 in 2007.

For HIV reports, currently about 70% of regional allocations of cases are based on patient postal code, 20% on health care provider postal code and 7% on laboratory postal code. For syphilis reports, 88% of allocations are based on patient postal code, 10% on health care provider, and less than 2% on laboratory postal code.

### Regional size of MSM populations

Absolute total size and regional distribution of MSM populations and HIV positive subpopulations

**Figure 1:**
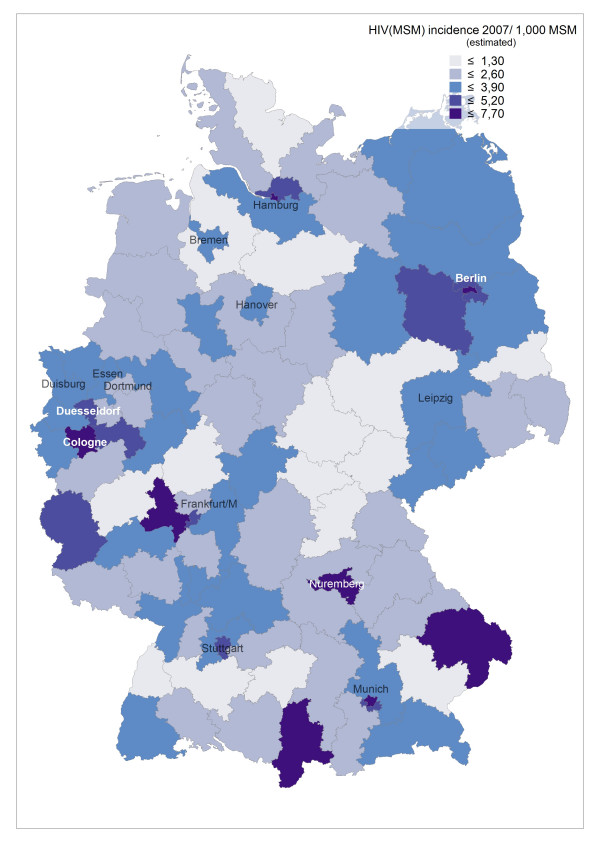
**Estimated incidence of newly diagnosed HIV in MSM per 1,000 MSM in 2007 in postal code regions of Germany**.

**Figure 2:**
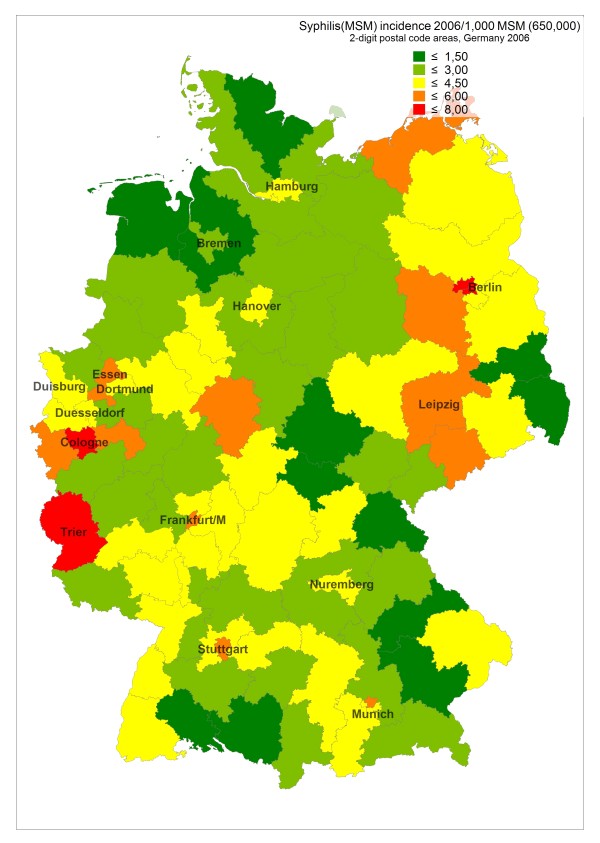
**Estimated incidence of newly diagnosed syphilis in MSM per 1,000 MSM in 2006 in postal code regions of Germany**.

**Figure 3:**
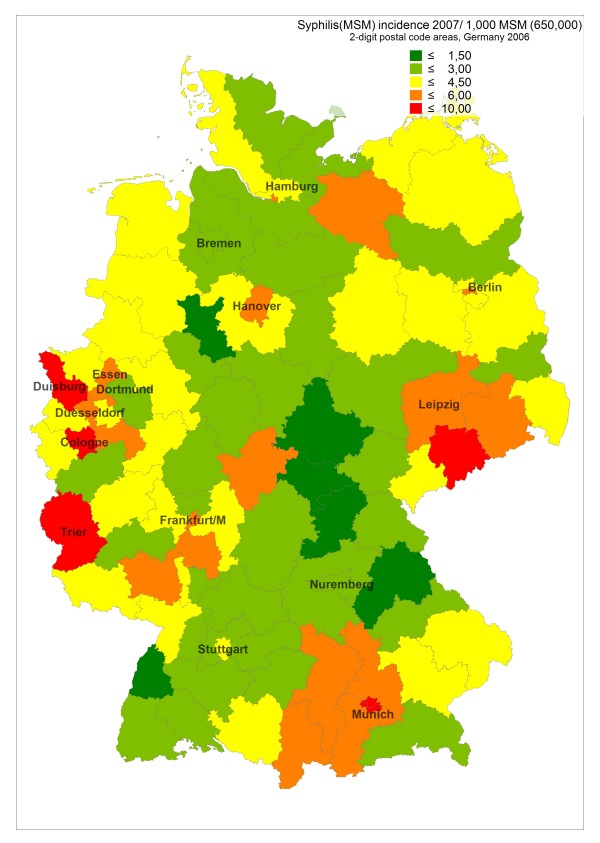
**Estimated incidence of newly diagnosed syphilis in MSM per 1,000 MSM in 2007 in postal code regions of Germany**.

The proportion of MSM in the adult male population in Germany was estimated from data on sexual preference collected in a representative telephone survey with 3,100 adult male participants in late 2007. This survey was conducted by the Federal Agency for Health Promotion (Bundeszentrale für gesundheitliche Aufklärung) for regular evaluation of HIV related health promotion activities. 2.5% (95%CI 1.5 – 3.4) of the male participants reported sexual contacts with men in the previous 12 months [[8], *BZgA, personal communication*]. Since general population surveys usually rather under- than overestimate homosexual contacts [9], which is still a stigmatized behaviour, we believe that the upper range of the confidence interval may reflect a more realistic range. Based on this proportion and population statistics provided by the Federal statistics agency (Statistisches Bundesamt), we estimated the number of MSM in the adult population between 20 and 59 years of age in Germany at approximately 575,000 to 785,000 persons (i.e. 2.5 – 3.4% of the adult male population). For incidence calculations in Additional file 1 and Figures 1, 2, and 3, we assumed a total MSM population of 650,000, resembling a MSM proportion of 2.9%.

The relative regional distribution of MSM – as well as of HIV positive MSM – in Germany was estimated based on the relative regional distribution of internet survey participants of MSM behaviour surveys, as described elsewhere [5]. We used the mean value of the relative proportion of survey participants, resp. HIV positive survey participants, from two separate surveys, the KABaSTI-study with n = 5,928 (n = 403 HIV positive) participants conducted in 2006 and the latest GMA-study with n = 8,170 (n = 545 HIV positive) participants conducted in 2007.

Proportional regional distribution of survey participants and the total MSM population estimate for Germany were used to calculate the absolute size of regional MSM populations.

The KABaSTI study was approved by the ethical committee of the Charité University Clinic in Berlin.

### Regional incidence of newly diagnosed HIV infection and syphilis in MSM populations

Using reports of newly diagnosed HIV infection and syphilis in MSM from the statutory surveillance system as numerator and the estimated regional MSM population as denominator we calculated MSM-specific incidence rates of newly diagnosed HIV infection and syphilis. Numbers for HIV and syphilis both include risk re-distribution of reports with missing information on transmission risk, and HIV numbers in addition include an adjustment for unrecognized multiple reports. Incidence rates were calculated for all 95 postal code regions defined by the first two digits of the five digit postal code. The largest cities are defined by one, two (Hamburg, Munich) or four (Berlin) two-digit postal code regions.

### MSM concentration factors

MSM concentration factors were calculated from both surveys for all 95 postal code regions by dividing the proportion of survey participants living in the respective region with the proportion of the male general population living in the region.

### Comparison of HIV prevalence estimates for regional MSM populations from models based on surveillance and on survey data

The number of MSM living with HIV in different regions was estimated by two methods. The surveillance data based method [10] is composed of back-calculation of incident HIV cases for the period 1980 until 1990 from AIDS cases reported up to 1995 [11], the number of newly diagnosed HIV infections from 1995 through 2008, including risk re-distribution of cases with missing information on transmission risk and adjustments for double reporting, as a surrogate for the number of incident infections during this period, and an interpolation of incident HIV cases from 1991 through 1994. This model does not consider changes of residence after HIV diagnosis and assumes a comparable regional distribution of as yet undiagnosed HIV infections in MSM.

The survey based method uses the mean of the proportional regional distribution of HIV positive participants from both surveys and the estimate of the total number of HIV positive MSM living in Germany from the surveillance data based model to estimate the regional size of the HIV positive MSM population.

## Results

### MSM-specific regional incidences of newly diagnosed HIV infections and syphilis

Incidence rates of newly diagnosed HIV infections in 2006 and 2007 remained highest in the largest cities (around 5–6/1,000 MSM in metropolitan areas), but incidence can be as high in surrounding regions and occasionally also in peripheral regions (see Figure 1, based on HIV surveillance data from 2006, and Additional file 1). E.g., the high incidence in border regions in the West, South, and Southeast of Germany reflect transient HIV incidence peaks related at least partly to syphilis outbreaks in these regions. Syphilis incidence showed a more diverse pattern, reflecting regional outbreaks within periodic epidemic waves sweeping through the MSM population (see Additional file 1, Figure 2 and 3, based on syphilis surveillance data from 2006 and 2007). Epidemiological trends for newly diagnosed HIV infections and syphilis among MSM in postal code regions over time (calculated for the period 2001–2007) usually reflect respective trends in the nearest gay centre (cities with larger MSM populations and a gay infrastructure of bars, discos and bathhouses; data not shown).

**Figure 4:**
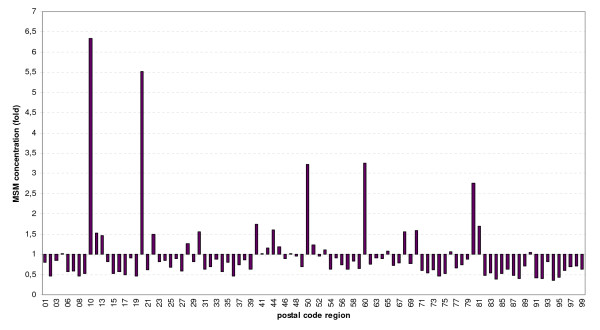
**Estimated relative regional distribution of the MSM population in postal code regions of Germany**.

**Figure 5:**
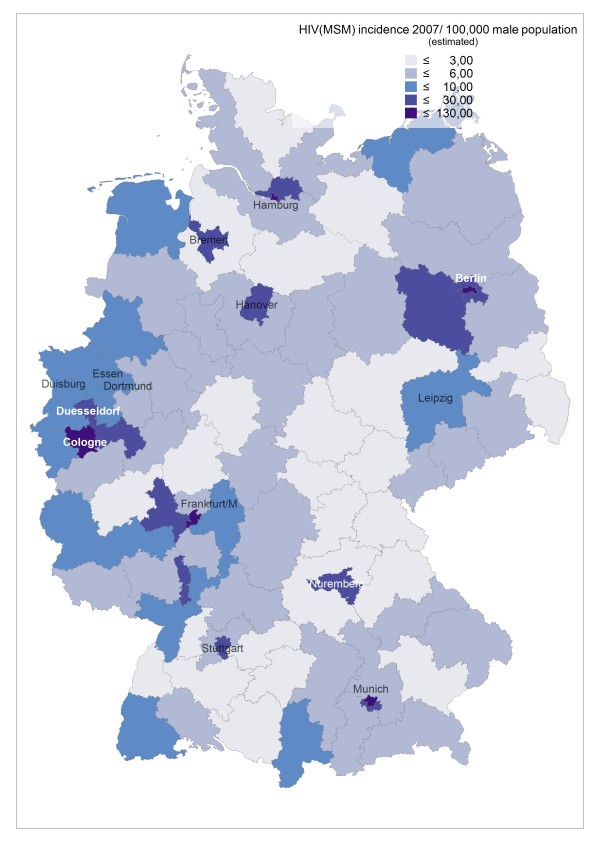
**Estimated incidence of newly diagnosed HIV in MSM per 100,000 men of the general population in 2007 in postal code regions of Germany**.

Due to the variable concentration of MSM in metropolitan areas (for MSM concentration factors in postal code areas resembling the largest cities of Germany see Figure 4), estimates for regional incidences of newly diagnosed HIV infections and syphilis with the total population or the total male population as denominator overestimate the disease burden of metropolitan MSM compared to MSM residing in non-metropolitan areas (see Figure 5 and Additional file 1). If the estimated MSM population is used as the denominator, HIV and syphilis incidence among MSM still show a gradient between metropolitan and rural areas. The gradient, however, becomes much smaller.

### Estimating regional HIV prevalence in MSM

The national HIV prevalence in MSM in 2008 was estimated to be 38,700 HIV infections [10]. If all these infections among an estimated number of 575,000 MSM in the age group 20–59 years (resembling a proportion of 2.5% MSM in the adult male population), the national HIV prevalence rate in MSM in Germany in this age group would be 6.7%. If we assume, that 3.4% of the adult male population are MSM, the HIV prevalence would decline to 4.9%. In Additional file 2, regional HIV prevalence rates in the estimated MSM populations of the 16 federal states of Germany and of the cities with the largest MSM populations are calculated for a proportion of 2.5% MSM in the 20–59 years old male general population (= 575.000 MSM, with a national HIV prevalence rate of 6.7%) and a proportion of 3.4% (= 785.000 MSM, with a national HIV prevalence rate of 4.9%. The estimates derived from survey data are higher than those based on surveillance data in all Eastern German states, particularly in Berlin, and lower in almost all Western German states, except Lower Saxony and Saarland, where the estimates are the same. In the larger cities, where MSM are concentrated, the range of the estimated HIV prevalence among MSM is between 5% and 16%, depending on the assumptions about the absolute size of the MSM population. If the mean values for the estimate ranges are compared, HIV prevalence in cities with the most developed infrastructure of gay venues and highest MSM concentrations ranges between 10 and 12%.

## Discussion

Using estimated MSM populations as denominator we observe much narrower ranges of HIV incidence rates (and incidence of other STI) between cities and federal states than suggested by incidence rates based on general population denominators. This indicates the advantage of this approach, because comparability of epidemiological data between different regions is improved.

An explanation for the moderate differences in incidence rate estimates for metropolitan and non-metropolitan MSM may be the high partner seeking mobility in MSM populations. MSM from rural areas and smaller cities often seek sexual partners outside of their place of residence, often in the next gay centre. Thus, the likelihood to meet sexual partners infected with HIV and/or syphilis may not be too different – at least in a country like Germany with a very well developed traffic and public transport infrastructure, which allows MSM in many non-metropolitan and rural areas to reach the next gay centre within less than two hours. In recent years, the use of the internet for finding sex partners has become a highly plausible additional factor for reducing differences between metropolitan and rural areas.

If we compare the estimated incidence of newly diagnosed HIV infections among German MSM (mean 0.3%, peak values in metropolitan areas around 0.5–0.7%) with HIV incidence among MSM in other types of studies in the Netherlands, we find rates in the same order of magnitude, especially if a participation bias towards sexually more active MSM outside of regular relationships in those studies is considered. In prospective cohort studies in MSM in Amsterdam and Rotterdam incidence rates between 1.1 and 1.5/100 person-years were observed between 1999 and 2005 [2].

In most, but not all cities and regions, incidence of newly diagnosed Syphilis in 2007 was higher than incidence of newly diagnosed HIV infection. Outbreak-like incidence peaks even higher than in metropolitan areas can occasionally be observed in peripheral regions like Trier (border region in the Western part of Germany; see Figure 2/3, Additional file 1).

We also analyzed whether it is feasible to estimate regional prevalence of HIV infections among MSM. Direct regional HIV prevalence estimates for MSM populations in all 95 postal code regions of Germany based on participation rates of self-reported HIV positive MSM in the Internet surveys are however not reliable due to the relatively small sample sizes of approximately 403 HIV positive participants in the KABaSTI and 545 in the GMA-2007 survey, which are distributed across 95 postal code areas. To minimize inaccuracies and biases due to low numbers per area, we evaluated to which extent the relative proportion of HIV positive MSM residing in the gay centres and in the 16 federal states may be reflected by the regional distribution of KABaSTI and GMA-2007 survey participants.

When the regional prevalent HIV cases in MSM are estimated according to the cumulative incidence of HIV reports in MSM adjusted for the estimated number of deaths between 1980 and 2008 (RKI prevalence model), the estimated HIV prevalence compared to the prevalence estimate based on the distribution of HIV-positive survey participants is higher in all western German federal states, and considerably lower in Berlin, Saxony, and the other federal states in Eastern Germany.

The main reason for this difference is the change of the epidemiological dynamics during the German reunification in 1990. While the cumulative distribution of HIV from the RKI model also reflects the regional distribution of HIV infections among MSM during the first wave of HIV infections in the 1980s, prevalence estimates based on survey participant distribution rather mirrors a distribution of currently sexually active MSM, and thus neglects infections which occurred many years ago. These infected persons may still be alive, but meanwhile sexually less active or not using the internet to find partners. This is especially relevant for the discrepancies observed between Western and Eastern Germany. The German Democratic Republic (Eastern Germany) had not experienced the first wave of HIV infections in the 1980s. Thus, after the German reunification in 1990, MSM in the eastern part of Germany and East-Berlin had a much lower HIV prevalence than MSM in Western Germany and West-Berlin in the early 1990s.

On the other hand, the surveillance data based method probably underestimates the prevalent cases in eastern German MSM, because for Eastern Germany the estimates are predominantly based on the number of already diagnosed infections, while in Western Germany a proportion of as yet undiagnosed infections is included in the model by using the back-calculation method for the early period of 1980 until 1990 (back-calculation based on AIDS cases accounts also for undiagnosed HIV cases). Thus, the real prevalence in MSM in Eastern Germany may lie somewhere in between the two estimates.

Other factors that may explain some of the differences between the surveillance data and survey based estimates:

1) Selective migration of HIV-positive MSM after HIV diagnosis from rural areas to larger cities and between cities (net gains to be expected especially for Berlin, Frankfurt and Leipzig, net losses for rural areas in Baden-Wuerttemberg, Bavaria, Lower Saxony, Rhineland-Palatina, Schleswig-Holstein, Mecklenburg-Vorpommern, Saxony-Anhalt, Thuringia and the cities Stuttgart and Hanover).

2) Underrepresentation of (HIV positive) MSM among the survey participants from the respective state/city (especially for Bavaria, where a difference between MSM population estimates based on proportion of MSM website user profiles and the proportion of survey participants has been described [5])

3) Overestimation of the proportion of HIV positive men living in a city by geographical attribution based on the postal code of the health care provider in the surveillance data based model (may be relevant especially for cities with large catchment areas in densely populated areas, such as Hamburg, Munich, Frankfurt, Duesseldorf and Stuttgart). In our experience the distinction between health care provider and patient postal code is not always reliable, thus a larger proportion of allocations than currently acknowledged may be based on the health care provider postal code.

The discrepancy between the two estimates disappears for the eastern German states except Berlin and becomes smaller for the western German states if we make a tentative adjustment in the survey based model for the federal states in Eastern Germany and reduce the prevalence estimate by 50%. Due to a later starting point of the HIV epidemic in MSM who live in the region of the former German Democratic Republic an adjustment of the estimate is justified. A 50% reduction could be justified by the fact that at the time of German reunification approximately 50% of the total cumulative HIV cases in Germany had already occurred in the former Western part of the country, and the number of prevalent cases would have a considerable impact on the number of new infections occurring in the period after the reunification. For the united federal state of Berlin which is geographically located in the eastern part of the country but is composed of the former West-Berlin (2.1 Mio. inhabitants) and the former East-Berlin (1.3 Mio. inhabitants), such kind of adjustments are more difficult. While MSM in West-Berlin had a similar or even higher HIV prevalence as other large cities in Western Germany, HIV prevalence in MSM in East-Berlin was dramatically lower before reunification. How much the estimate for Berlin based on the observed prevalence among survey participants could be reduced to account for the "reunification effect" is unclear. However, it does not seem realistic to explain the large difference between survey and surveillance based estimates for Berlin by such a "reunification effect".

For the relative proportions of the federal states of Bavaria and North Rhine-Westphalia some adjustments might be reasonable as well because we observed a slightly skewed representation of survey participants from these two states compared with the MSM website profile data from the largest German MSM website (GayRomeo): from the KABaSTI participants who were recruited on GayRomeo 14.6% reported residence in Bavaria, 19.3% residence in North Rhine-Westphalia, compared with 10.6% and 25% of all survey participants. But again, even if we adjust the data according to these proportions, the surveillance based estimate for Bavaria will remain higher than the survey based estimate.

In Germany, a major challenge for regional prevalence estimates arises from temporal changes of the spread of HIV in MSM populations, mainly from the different epidemiological dynamics in the Western and Eastern part of the country before reunification. Another factor which is difficult to assess is selective migration of HIV positive men to metropolitan areas after HIV diagnosis. As life expectancy and quality of life of people living with HIV have improved during the last decade, such selective migration may have played an increasing role in recent years. Because of the existence of larger sexual networks of HIV-positive MSM in metropolitan areas and because of better access to quality medical HIV care, MSM diagnosed with HIV infection in non-metropolitan areas may see even larger benefits from moving to metropolitan areas than their non-infected peers. The pronounced differences between the surveillance and survey based HIV prevalence estimates for Berlin and Bavaria may be an indication for such selective migration processes. Questions about post-HIV diagnosis migration of HIV positive MSM in clinical surveillance studies could be used to verify this hypothesis.

## Conclusion

The regional distribution of participants of internet convenience samples may be used as a tool to estimate the regional distribution of a "hidden" population like MSM. Potential biases should be considered, which may arise from subtle differences in regional participation rates and recruitment on websites with skewed user characteristics. Together with data on reported transmission group from national infectious disease surveillance systems and with total population size estimates for MSM, local or regional population group-specific incidence and prevalence for HIV and incidence for syphilis and other STIs can be estimated. Compared with infectious disease surveillance based models for the estimation of regional HIV prevalence, the estimates based on internet survey data may be able to reflect post-HIV diagnosis migration of HIV positive MSM. Resulting estimates are within expected and plausible ranges and could be used to compare regional epidemiological trends, prevention needs, and efficacy of prevention activities.

## Competing interests

The authors declare that they have no competing interests.

## Authors' contributions

UM conceived and designed the KABaSTI study, OH designed the statutory surveillance system for HIV and syphilis. The KABaSTI and GMA survey were coordinated by AJS, data analysis and interpretation were done by UM and AJS. The surveillance data based model was developed by OH and updated and revised by UM and CK, with supervision by OH. The idea for linking and comparing regional distribution of survey participants with statutory surveillance data came from UM, AJS compared the KABaSTI sample with the GMA sample. The manuscript was drafted by UM and AJS and critically revised by OH. All authors read and approved the final manuscript.

## Pre-publication history

The pre-publication history for this paper can be accessed here:



## Supplementary Material

Additional file 1 **Incidence of newly diagnosed HIV infection (n = 2,180) and syphilis (n = 2,622) in the MSM transmission group in 2007**. Incidence estimates for federal states and the largest cities are calculated per 100,000 male population and compared to estimated incidence of newly diagnosed HIV infection and syphilis per 1000 estimated MSM population (assuming a total number of 650,000 MSM) Click here for file

Additional file 2 **Estimates for the regional distribution of MSM and HIV prevalence by end of 2008 in Federal States in Germany**. Estimation of the MSM population size, HIV prevalence, and HIV prevalence rates among MSM for all federal states and largest cities of Germany Click here for file
