# Supplementary material for: The denominator problem: Estimating MSM-specific incidence of sexually transmitted infections and prevalence of HIV using population sizes of MSM derived from Internet surveys
Source: BMC Public Health. 2009 Jun 11;9:181. doi: 10.1186/1471-2458-9-181 (PMC2702384; doi:10.1186/1471-2458-9-181)
Supplement: Additional file 1 — Incidence of newly diagnosed HIV infection (n = 2,180) and syphilis (n = 2,622) in the MSM transmission group in 2007. Incidence estimates for federal states and the largest cities are calculated per 100,000 male population and compared to estimated incidence of newly diagnosed HIV infection and syphilis per 1000 estimated MSM population (assuming a total number of 650,000 MSM) [file 1471-2458-9-181-S1.doc]

**Additional file 1: Incidence of newly diagnosed HIV infection (n=2,180) and syphilis (n=2,622) in the MSM transmission group in 2007***

| Postal code area | *HIV(MSM)/100,000 male population* | HIV/1,000 MSM  (survey-based estimate for regional population size) | *Syphilis (MSM) / 100,000 male population* | Syphilis/1,000 MSM |
| --- | --- | --- | --- | --- |
| 04 Leipzig | *8.0* | 2.7 | *14.5* | 4.9 |
| 10 Berlin | *103.4* | 5.7 | *103.8* | 5.7 |
| 12 Berlin | *18.5* | 4.2 | *13.6* | 3.1 |
| 13 Berlin | *19.4* | 4.6 | *18.0* | 4.3 |
| 14 Berlin/Potsdam | *10.8* | 4.6 | *8.4* | 3.6 |
| 20 Hamburg | *120.9* | 7.6 | *84.4* | 4.3 |
| 22 Hamburg | *20.2* | 4.7 | *15.5* | 3.6 |
| 30 Hanover | *13.3* | 3.0 | *21.2* | 4.7 |
| 31 Hanover surroundings | *3.2* | 1.7 | *5.7* | 3.1 |
| 40 Dusseldorf | *24.6* | 4.9 | *27.7* | 5.5 |
| 45 Essen | *9.8* | 2.9 | *16.3* | 4.8 |
| 50 Cologne | *50.6* | 5.5 | *58.7* | 6.4 |
| 54 Trier | *8.7* | 4.8 | *16.6* | 9.2 |
| 60 Frankfurt | *38.7* | 4.1 | *55.6* | 6.0 |
| 65 Wiesbaden | *20.0* | 6.5 | *10.9* | 3.5 |
| 68 Mannheim | *10.2* | 3.2 | *15.4* | 3.5 |
| 70 Stuttgart | *23.2* | 5.1 | *20.3* | 4.5 |
| 71 Stuttgart surroundings | *5.7* | 3.3 | *4.8* | 2.8 |
| 80 Munich | *48.6* | 6.1 | *66.0* | 8.3 |
| 81 Munich | *21.6* | 4.4 | *38.2* | 7.9 |
| 90 Nuremberg | *17.0* | 5.6 | *7.0* | 2.3 |

*Incidence estimates calculated per 100,000 male population are compared to estimated incidence of newly diagnosed HIV infection and syphilis per 1000 estimated MSM population (assuming a total number of 650,000 MSM) by postal code areas in Germany.

The estimates for the regional MSM population size (denominator) show the same range as for prevalence estimates (see Additional file 2). The numerator, which is composed of the number of newly diagnosed and reported cases of HIV and syphilis from the respective region, should be seen only as a proxy for the actual incidence due to a range of different confounding factors: time of diagnosis does not necessarily reflect time of infection; place of diagnosis does not necessarily reflect place of residence or place of transmission; number of reported cases may be different from number of diagnosed cases due to underreporting or failure to identify a reported case as newly diagnosed. Due to this multitude of potential confounders we decided, not to present ranges for these estimates, which should be seen as what they are: crude estimates, which are useful to make comparisons between cities and regions over time but which can not claim to exactly describe infection incidence in a certain region and period.
