# Supplementary material for: The denominator problem: Estimating MSM-specific incidence of sexually transmitted infections and prevalence of HIV using population sizes of MSM derived from Internet surveys
Source: BMC Public Health. 2009 Jun 11;9:181. doi: 10.1186/1471-2458-9-181 (PMC2702384; doi:10.1186/1471-2458-9-181)
Supplement: Additional file 2 — Estimates for the regional distribution of MSM and HIV prevalence by end of 2008 in Federal States in Germany. Estimation of the MSM population size, HIV prevalence, and HIV prevalence rates among MSM for all federal states and largest cities of Germany [file 1471-2458-9-181-S2.doc]

## Additional file 2: Estimates for the regional distribution of MSM and HIV prevalence by end of 2008 in Federal States in Germany

| German Federal States and *largest cities*, in alphabetical order  (postal code areas) | MSM total (20-59 y)  Regional distribution based on mean value from KABaSTI 2006 study and GMA-2007 study | | Regional HIV-prevalence in MSM estimated according to proportional distribution of HIV positive survey participants | Regional HIV prevalence in MSM estimated according to surveillance data based model | Estimated range of HIV prevalence rate in MSM (20-59y) if all HIV positive men were in the age group 20-59 y |
| --- | --- | --- | --- | --- | --- |
|  | 2.5% MSM | 3.4 % MSM | Number of MSM living with HIV in Germany  Range: 37,100 – 40,300 | |  |
| Baden-Wuerttemberg  (68 – 79, 88, 89) | 62,200 | 85,000 | 2,200 – 2,400 | 2,600 – 2,900 | 2.6 – 4.7% |
| *Stuttgart*  *(70)* | *9,100* | *12,500* | *550 - 600* | *n.a.* | *4,4 – 6.6%* |
| Bavaria  (80 – 87, 90 -97) | 62,600 | 85,400 | 3,600 – 3,900 | 5,600 – 6,100 | 4.2 – 9.7% |
| *Munich*  *(80 – 81)* | *20,650* | *28,200* | *2,100 – 2,250* | *n.a.* | *7.4 – 10.9%* |
| Berlin  (10 – 14) | 77,000 | 105,000 | 10,000 – 10,900 | 6,400 – 6,900 | 8.2–14.2% |
| Brandenburg  (14 – 16) | 6,900 | 10,000 | 330 - 350 | 200 - 230 | 2.0 – 5,1% |
| Bremen  (28) | 6,450 | 8,800 | 470 - 510 | 550 - 600 | 5.3 – 9.3% |
| Hamburg  (20,22) | 25,500 | 34,500 | 2,860 - 3,100 | 3,900 - 4,200 | 8.2 – 16.5% |
| Hesse  (34-36, 60 – 65) | 47,500 | 65,000 | 3,500 – 3,800 | 3,900 - 4,100 | 5.4 – 8.6% |
| *Frankfurt*  *(60 – 61)* | *16,000* | *22,000* | *1,900 - 2,100* | *n.a.* | *8.6 – 13.1%* |
| Mecklenburg-Vorpommern  (17 - 19) | 8,000 | 11,000 | 310 - 340 | 110 - 130 | 1.0 – 4.3% |
| Lower Saxony  (21,26,27,29-31, 37,38,49) | 49,500 | 67,700 | 2,200 – 2,400 | 2,200 – 2,400 | 3.2 -4.8% |
| *Hanover*  *(30)* | *10,000* | *13,500* | *550 - 600* | *n.a.* | *4.0 – 6.0%* |
| North Rhine- Westphalia  (32, 33, 40-48, 50 - 53) | 148,000 | 202,500 | 8,100 – 8,800 | 8,500 – 9,300 | 4.0 – 6.3% |
| *Cologne*  *(50)* | *25,500* | *34,800* | *2,000 – 2,100* | *n.a.* | *5.7 – 8.2%* |
| *Duesseldorf*  *(40)* | *12,000* | *16,400* | *950 - 1,000* | *n.a.* | *5.8 – 8.3%* |
| Rhineland-Palatina  (54 – 56, 67) | 17,200 | 23,500 | 820 - 900 | 1,000 - 1,100 | 3.5 – 6.4% |
| Saarland  (66) | 6,700 | 9,100 | 350 - 380 | 360 - 380 | 3.8 – 5.7% |
| Saxony  (01-04, 08,09) | 25,000 | 35,000 | 1,100 – 1,200 | 480 - 550 | 1.4 – 4.7% |
| *Leipzig*  *(04)* | *9,100* | *12,500* | *590 - 640* | *n.a.* | *? – 7.0%* |
| Saxony-Anhalt  (39, 06) | 9,800 | 13,400 | 350 - 370 | 160 - 180 | 1.2 – 3.8% |
| Schleswig-Holstein  (23-25) | 13,500 | 18,400 | 900 - 980 | 1,000 - 1,100 | 4.9 – 8.1% |
| Thuringia  (07, 98,99) | 8,900 | 12,200 | 235 - 255 | 120 - 140 | 1.0 – 2.9% |

n.a.= not available
